# Reconstructing the infrared spectrum of a peptide from representative conformers of the full canonical ensemble

**DOI:** 10.1038/s42004-023-00835-3

**Published:** 2023-03-03

**Authors:** Amir Kotobi, Lucas Schwob, Gregor B. Vonbun-Feldbauer, Mariana Rossi, Piero Gasparotto, Christian Feiler, Giel Berden, Jos Oomens, Bart Oostenrijk, Debora Scuderi, Sadia Bari, Robert H. Meißner

**Affiliations:** 1 Deutsches Elektronen-Synchrotron DESY, Hamburg, Germany; 2 Hamburg University of Technology, Institute of Advanced Ceramics, Hamburg, Germany; 3 Max Planck Institute for the Structure and Dynamics of Matter, Hamburg, Germany; 4 Scientific Computing Division, Paul Scherrer Institute, Villigen, Switzerland; 5 Helmholtz-Zentrum Hereon, Institute of Surface Science, Geesthacht, Germany; 6 Radboud University, Institute for Molecules and Materials, FELIX Laboratory, Nijmegen, The Netherlands; 7 The Hamburg Centre for Ultrafast Imaging, Hamburg, Germany; 8 Institut de Chimie Physique, CNRS UMR8000, Université Paris-Saclay, Orsay, France; 9 Zernike Institute for Advanced Materials, University of Groningen, Groningen, The Netherlands; 10 Hamburg University of Technology, Institute of Polymers and Composites, Hamburg, Germany

**Keywords:** Chemical physics, Molecular modelling, Computational chemistry, Infrared spectroscopy, Peptides

## Abstract

Leucine enkephalin (LeuEnk), a biologically active endogenous opioid pentapeptide, has been under intense investigation because it is small enough to allow efficient use of sophisticated computational methods and large enough to provide insights into low-lying minima of its conformational space. Here, we reproduce and interpret experimental infrared (IR) spectra of this model peptide in gas phase using a combination of replica-exchange molecular dynamics simulations, machine learning, and ab initio calculations. In particular, we evaluate the possibility of averaging representative structural contributions to obtain an accurate computed spectrum that accounts for the corresponding canonical ensemble of the real experimental situation. Representative conformers are identified by partitioning the conformational phase space into subensembles of similar conformers. The IR contribution of each representative conformer is calculated from ab initio and weighted according to the population of each cluster. Convergence of the averaged IR signal is rationalized by merging contributions in a hierarchical clustering and the comparison to IR multiple photon dissociation experiments. The improvements achieved by decomposing clusters containing similar conformations into even smaller subensembles is strong evidence that a thorough assessment of the conformational landscape and the associated hydrogen bonding is a prerequisite for deciphering important fingerprints in experimental spectroscopic data.

## Abstract (web summary)

Infrared spectroscopy is widely used for detailed insights into the three-dimensional molecular structure of biomolecules, however, the accurate description of experimental data by theoretical approaches remains challenging due to the dynamic processes that occur in biomolecules. Here, the authors report the accurate interpretation and reproduction of experimental infrared spectra of a model peptide in the gas phase using a combination of replica-exchange molecular dynamics simulations, machine learning and ab initio calculations based on representative structural conformers.

## Introduction

Dynamic processes in biomolecules, such as secondary structural changes in peptides and proteins, pose a challenge for the accurate and quantitative description of experimental data by theoretical approaches. Several structure-determining factors, such as the intramolecular hydrogen-bonding (H-bonding) pattern, play an important role in the rapid transformation between structural motifs. Moreover, the coupling between different vibrational modes lead to anharmonicity and complex intra- and intermolecular energy exchanges^1^. Although parts of these intricate relationships can be explained by the conformational diversity evident in all measured spectra, as shown in this work, it should be kept in mind that this still cannot lead to perfect agreement between experiment and theory, since anharmonicities (i.e., combination bands, overtones, and Fermi resonances) are difficult to treat even with very sophisticated theoretical approaches.

A useful approach to gain a fundamental understanding of these processes is to predict theoretical spectra and relate those to experimental measurements^2^. However, to facilitate direct validation of theoretical predictions, solvation effects are often neglected or considered by frequency maps^3,4^ and predictions are usually made on isolated systems. Frequency maps, either from ab initio^5^, empirical^6^ or using machine learning^7^, correct the vibrational frequencies for solvent effects by using maps connecting the vibrational frequency shifts with the local electrostatic environment. Frequency maps have also been successfully used to correct vibrational frequencies for intermolecular H-bonding, although to our knowledge only for a rather simple system of liquid water^8^. Thus, although the link between gas-phase studies and biologically relevant reactions is discussed controversially, many important properties (e.g., protonation or interactions with ions and solvation effects) can be studied in gas-phase experiments, allowing a comparison with theoretical methods, such as first-principles and molecular mechanics approaches^9–11^.

Investigating different secondary structure motifs in the gas phase is thus a valuable approach that eases the investigation of structure-property relationships and helps disentangling the delicate balance between enthalpic and entropic contributions in an unperturbed environment^9,10^. In this regard, electrospray ionization (ESI)^12^ gives the opportunity to bring molecular ions into solvent-free gas phase in which the native-like conformations in the absence of solvent remains intact^13^. In addition, several experimental studies based on the combination of mass spectrometry and infrared (IR) spectroscopy have been proposed for revealing the secondary structures of charged gas-phase peptides and proteins^14–16^. Among gas-phase experimental techniques, IR spectroscopy is widely used as it provides detailed insights into the three-dimensional molecular structure, as well as into intra- and intermolecular interactions of gas-phase protein ions^15,17–19^ and polymers^20^. However, the low free-energy barriers between conformations and the vast configuration space of even relatively small peptides, especially for intrinsically disordered proteins and peptides, result in overlapping signals from the many nonspecific, i.e. nonhelical or sheet-like, conformations commonly encountered in experimental measurements^21,22^. Addressing discrepancies between theory and experiments has been carried out in several studies using different organic materials^23–26^. As we will demonstrate, considering only a few individual conformations in theoretical predictions without taking into account their correct statistical ensemble weight (which is common practice in computational spectroscopy) can hamper the interpretation of experimental results and lead to discrepancies between theory and experiment.

In this study, we explore the structural landscape of leucine enkephalin (LeuEnk), an experimentally well-studied biologically active endogenous opioid pentapeptide, by combining Replica-Exchange Molecular Dynamics (REMD) simulations with machine learning. LeuEnk is a well-established standard in ESI-based biomolecular mass spectrometry^27,28^ and has been investigated extensively using IR-UV double resonance photofragment spectroscopy to obtain conformer-specific spectra at cryogenic temperatures^14,26,29,30^, as well as infrared multiphoton dissociation (IRMPD) at room temperature^31,32^. LeuEnk lies here in an interesting size regime: small enough to efficiently use sophisticated computational methods while, on the other hand, large enough to allow insights into the low-lying minima of the conformational space^33,34^ and competing H-bonded networks. IRMPD experiments have been extremely useful in understanding key features of theoretical predictions^24,29,35^.

In this study, comparisons with IRMPD experiments in the gas phase are performed based on representative peptide conformations identified from extensive REMD simulations and clustering. IR spectra were calculated at ab initio levels for each identified representative conformer. The influence of the conformational ensemble was assessed by means of a hierarchical clustering using the Probabilistic Analysis of Molecular Motifs (PAMM)^36^. While spectra from LeuEnk were experimentally measured in the gas phase at room temperature using IRMPD, the above-mentioned methods were used to study the conformational averaging of recurring motifs to finally predict the theoretical IR spectrum at a finite temperature with high accuracy.

## Results and discussion

**Fig. 1:**
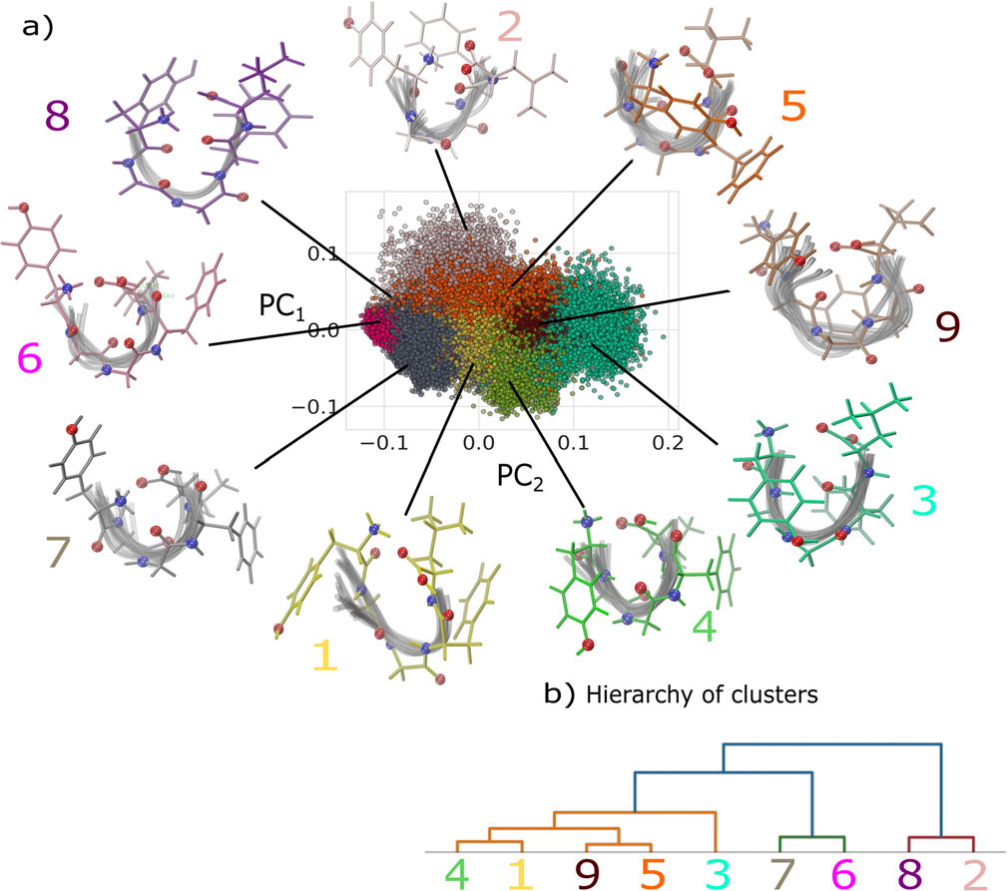
Low-dimensional conformer map of the gas-phase LeuEnk SOAP feature space at 300 K. The first two Principal Components (PC) of a Principal Component Analysis (PCA) and the classification identified using PAMM are shown (**a**). More details on the PCA are found in the SI. Colored molecular line representations correspond to the most energetically favorable (i.e. representative) conformer of each cluster, while the gray-shaded configurations were randomly selected from the same cluster and shown for comparison. Hierarchy of clusters (**b**) illustrating the similarities of clusters as they result from hierarchical clustering to facilitate grouping of similar conformer clusters. Color coding corresponds to molecular conformers and to the dots in the low-dimensional conformer map in **a**.

We used a Principal Component Analysis (PCA) to project the entire structural landscape of an N-terminal protonated LeuEnk (amino acid sequence [YGGFL+H]^+^) shown in Supplementary Fig. S1, sampled during the REMD at the experimental relevant temperature of 300 K, on the basis of the first two principal components of the Smooth Overlap of Atomic Positions (SOAP) feature space (cf. Supplementary Fig. S2), which results in an easily interpretable two-dimensional map given in Fig. 1a, where points are colored additionally according to the PAMM cluster they belong to. The conformer with the lowest energy of each cluster (cf. Supplementary Fig. S3) is considered as the representative motif of the corresponding cluster and those are shown around the map. The dendrogram in Fig. 1b shows the hierarchical merging of conformer clusters and the structural metastability represented by this dendrogram reveals the connection between the free-energy basins (and associated conformers). The colors correspond to the coloring of the molecules in Fig. 1a and are intended to visually help to group similar conformer clusters together. The hierarchical merging is done in a controlled way by checking how fuzzy the cluster boundaries are (see ref. ^36^ for more information on how the merging of clusters is achieved). As in reality biomolecules actively explore the free-energy landscape continuously^37^, a weighting factor *w*, which estimates the statistical relevance of the various metastable states is introduced. *w* resembles the canonical ensemble weight and is obtained from the population of structures within a cluster normalized with the overall population and is given in percentage in Table 1. Remarkably, cluster **7**, the conformer with the highest relevance, is also the one identified in a previous work of Burke et al.^14,29^ in terms of its secondary structure and H-bond configuration. While cluster **5**, the cluster with the second highest relevance, is quite similar to cluster **7** in its secondary structure, the side-chain angles differ (a detailed conformer comparison of both clusters is shown in Supplementary Fig. S5). It should be noted that the selection of the representative conformer via the lowest REMD energy also appears to robustly identify the corresponding geometry optimized ab initio conformer with comparably low energy, as discussed in the SI.

**Table 1:** Summary of LeuEnk H-bonding.

Cluster	〈*s*_A_〉	〈*s*_D_〉	Most probable (*s*_A_, *s*_D_)	*w*%
**1**	1.82 ± 0.64	1.69 ± 0.78	(1,1) 49%	13.0%
**2**	1.71 ± 0.56	1.92 ± 0.74	(1,3) 52%	4.6%
**3**	1.41 ± 0.26	1.67 ± 0.58	(1,1) 37%	9.8%
**4**	1.54 ± 0.40	1.69 ± 0.57	(1,1) 40%	14.2%
**5**	1.59 ± 0.42	1.77 ± 0.48	(1,2) 30%	20.1%
**6**	1.53 ± 0.40	1.65 ± 0.41	(1,1) 34%	2.5%
**7**	1.34 ± 0.29	1.74 ± 0.31	(1,2) 42%	29.3%
**8**	1.95 ± 0.01	0.95 ± 0.01	(2,1) 60%	0.2%
**9**	1.67 ± 0.42	1.82 ± 0.58	(2,1) 30%	6.3%

〈*s*_D_〉 and 〈*s*_A_〉 denotes the weighted averaged number of donated and accepted NH⋯O and its standard deviation. *w* quantifies the weight of each cluster with respect to the relative population of each cluster to the total number of conformers found in the REMD trajectory at 300 K. Most probable (two-dimensional) H-bonding configurations are given as tuples along with their joint probability.

### Hydrogen-bonding statistics

**Fig. 2:**
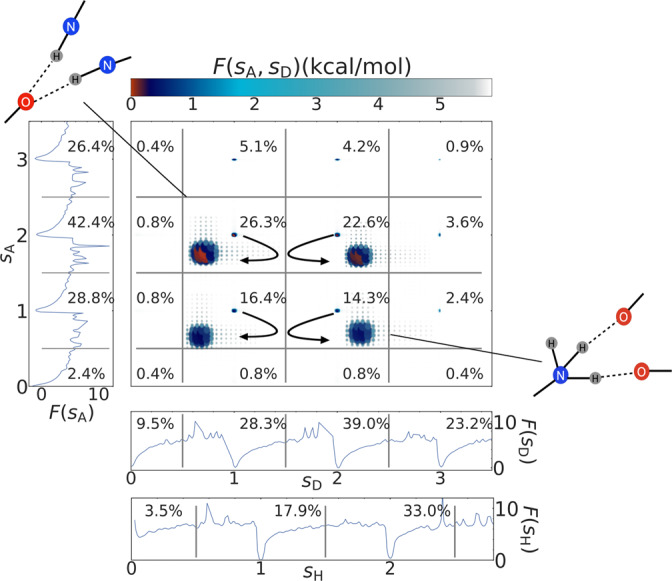
Hydrogen-bonding statistics of LeuEnk from the REMD trajectory at 300 K. Probability distributions have been smoothed with triangular kernel of width 0.025 and are represented in terms of $F=-{k}_{{{{{{{{\rm{B}}}}}}}}}T\ln (P)$, expressed in kcal/mol. We also report the integrated (joint) probabilities (in percent) for the corresponding region of different integer values of *s*_A_, *s*_D_, and *s*_H_. Schematic representations of two exemplary H-bonding configurations are given in the insets.

In general, the REMD simulation showed highly dynamic changes in the H-bonding patterns throughout the simulation. Figure 2 shows the H-bonding free energies of N−H⋯O at 300 K obtained from the full REMD trajectory at that temperature. As will be explained a little later, N−H⋯O is the predominant H-bonding motif in the gas phase of LeuEnk where no water is present. In Fig. 2, *s*_A_ and *s*_D_ denote the number of H-bonding accepted and donated in N−H⋯O triplets, while *s*_H_ is the number of hydrogen bonds in which a hydrogen is involved. More specifically, the H-bonding definition provided by PAMM consists of a continuous function that given in input a specific triplet^38^ returns a real number between 0 and 1, with 1 being a perfect match with the typical H-bonding pattern found in the training data. Thus, summing and averaging over all the possible triplets in which a specific tagged atom is involved we can define a collective variable that effectively corresponds to an H-bonding counting function. In the example reported in Fig. 2, *s*_D_ is defined as the sum over all the possible triplets in which a generic tagged N is acting as donor in an N−H⋯O pattern. Similarly, *s*_A_ is defined as the sum of all the possible triplets in which a generic tagged O is acting as acceptor in an N−H⋯O pattern. *s*_A_, *s*_D_, and *s*_H_ are calculated for each snapshot of the REMD trajectory using the HBPAMM^38^ implementation from the PAMM package. The probabilities *P* for these values are obtained from the normalized and smoothed histograms of these quantities, i.e., the probability distributions, and consequently used to calculate the free-energy equivalent via $F=-{k}_{{{{{{{{\rm{B}}}}}}}}}T\ln (P)$, where *P* denotes an unbiased probability of the different H-bond configurations, as obtained from the histograms of *s*_A_, *s*_D_, and *s*_H_ calculated from the REMD trajectory at 300 K, while *k*_B_ is the Boltzmann constant and *T* is the temperature. Note that *s*_D_ indicates the number of hydrogens donated by nitrogen in general, independent of the specific nitrogen and the total number of nitrogens in the molecule (nitrogens that do not donate hydrogens are ignored in the calculations). This is similarly true for *s*_A_ and *s*_H_. A consequence of this definition is that whenever *s*_D_ is greater than 1, the N-terminal ${{{{{{{{{\rm{NH}}}}}}}}}_{3}}^{+}$ is definitely involved in H-bonding, since otherwise there are only NH groups in the molecule that can donate only a single hydrogen. On the other hand, if *s*_D_ is very close to two or even above, it means that basically only the ${{{{{{{{{\rm{NH}}}}}}}}}_{3}}^{+}$ group is involved in H-bonding (another possibility which would give *s*_D_ = 3 would be a rather unlikely configuration in which ${{{{{{{{{\rm{NH}}}}}}}}}_{3}}^{+}$ donates three hydrogen bonds while another single NH donates one). *s*_D_ = 0 means, obviously, that no H-bonding occurs in the N−H⋯O triplets.

Integration over probability distributions in the different H-bonding regions in Fig. 2 resulting in the mentioned percentages is used to explain the (often just subtle) differences between the identified PAMM clusters. Percentages reported in the free-energy surface of Fig. 2 denote the integrated joint probability of finding a configuration in the vicinity of the different integer numbers of donors and acceptors. The percentages in the free-energy profiles on the sides of Fig. 2 denote the integrated probabilities in the vicinity of the integer number of hydrogen bond acceptors (or donors), regardless of the number of donors (or acceptors, respectively). Consequently, the (averaged) H-bonding pattern of N−H⋯O, denoted by 〈*s*_D_〉 and 〈*s*_A_〉, and the most probable H-bonding configuration (*s*_A_, *s*_D_) along with its joint probability are calculated for each subensemble and are given for each identified cluster in Table 1 While the averaged H-bonding values 〈*s*_A_〉 and 〈*s*_D_〉 show some differences between subesemble clusters, the most probable H-bonding patterns (*s*_A_, *s*_D_) are more distinct and are quite different for each conformer indicating a more or less pronounced involvement of the ${{{{{{{{{\rm{NH}}}}}}}}}_{3}}^{+}$ group. Moreover, the observed high standard deviations of the averaged values are further evidence of the quite dynamic H-bonding observed during the REMD.

Both the N- and C-termini are usually involved in H-bonding and thus LeuEnk often resembles a *β*-hairpin or *α*-helix secondary structure motif. Clearly, the ${{{{{{{{{\rm{NH}}}}}}}}}_{3}}^{+}$ group plays an important role in forming these secondary structures due to the engagement of this group with several nucleophilic regions of the molecule. Interaction between amide hydrogens and carbonyls and the strong COO-H⋯O=C at the C-terminal are characteristic of *γ*-turns and noticeable interactions for most clusters. Although the involvement of the carboxyl or phenol in the formation of O−H⋯O hydrogen bonds is often of great importance, especially when water is nearby, we neglect this type of otherwise quite important H-bonding in our analysis for simplicity, since H-bonding of the type N−H⋯O is able to explain most of the conformer structural differences. Moreover, if there are O−H⋯O H-bonds (in cluster **2**, **3** and **9** this H-bonding is not apparent) they are always formed between the first and second glycine and the carboxyl and phenyl group, respectively. Thus, this type of H-bonding does not contribute to the highly dynamic H-bonding behavior. Motifs of the clusters **1** and **4**, which have lower probability, form usually *α*-helix-like motifs. However, the motif of cluster **3** contains probably a *δ*-turn, which is sterically quite improbable. It is worth pointing out that clusters **2**, **3** and **9** lack a strong COO-H⋯O=C at the C-terminal. While the carbonyl in the third glycine of LeuEnk shows typically just one H-bond with amides for most clusters, the motif of cluster **9** has interactions of this group with phenylalanine and leucine amides and an absent H-bonding between N- and C-terminus.

Overall, H-bonding strongly correlates with the wavenumber and intensity of different vibrational bands^39,40^. Thus, combining the information on the contribution of H-bonding in both the C=O and N–H groups to the overall IR bands in the following elucidates the impact of the H-bonding of the different conformers on the final IR signal.

### Infrared spectroscopy of identified recurring structural motifs

Armed with the information on the LeuEnk conformers and their H-bonding network, we are now in the position to evaluate the influence of the subensembles from the REMD simulation on the IR signatures of LeuEnk. It should be noted, however, that the broadening and shape intensity of the peaks in the experiment are influenced only partly by effects related to the conformational dynamics of the molecules^18^. One has to take into account that the formation of the lowest energy conformation is driven by a balance between kinetic, enthalpic, and entropic effects. The kinetic trapping, specific to the experimental conditions, can be entering into the final balance and cause the formation of different conformers^41^. The conformational distribution depends thus strongly on the thermodynamic conditions in the experiments as these have a strong impact on the formation pathway to the metastable conformations. Another issue that should be taken into consideration is that experimental IRMPD spectra are usually compared to calculated linear absorption spectra. In reality, IRMPD spectra depend on very complex mechanisms involving sequential photon absorption, stimulated and spontaneous emission, energy distribution, and fragmentation, all of which have their own time scales and will affect the peak shape^42^. Despite the theoretical complexity of the interpretation, it has been shown that IRMPD spectra agree with calculated ones if the comparison is performed carefully. Hence, it is generally accepted that the peak positions obtained from theory can be trusted^22^. As we show here, consideration of representative conformers can improve the IRMPD spectrum prediction significantly.

**Fig. 3:**
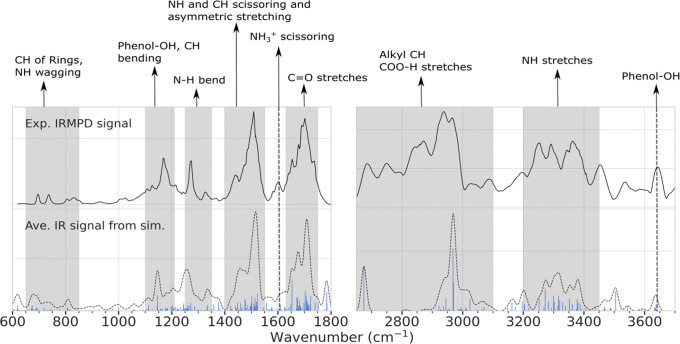
Comparison between experimental IRMPD and predicted average IR spectra using the PAMM conformers. Calculated IR intensities are convoluted by Gaussians with a 10 cm^−1^ full-width of half-maximum. Pendry reliability factors *R*_P_ are 0.56 for the left panel and 0.7 for the right panel.

In this study, harmonic vibrational modes of representative conformers for each identified PAMM cluster have been calculated in order to compare the vibrational modes of the obtained conformational families—represented by a single conformer—to experimentally observed spectroscopic fingerprints. It should be noted, however, that while this approach should be able to capture the main features of the vibrational spectra of the corresponding conformational family, some particular features may be missing as a result of neglecting other important local minima, anharmonicity, and other effects. Figure 3 shows the weighted average IR spectra of the amide I/II/III/V and amide A/B regions of the 9 PAMM conformers from Fig. 1 using their corresponding weighting factor and compares the prediction with the experimental IRMPD spectra along with Pendry reliability factors. Supplementary Table S3 reports the IR shift of amide I peak of PAMM representative conformations with respect to IRMPD experimental spectrum. Pendry reliability factors^43^ *R*_P_ are often used for unambiguous comparisons of theoretical and experimental IR spectra^22^ and are given in the caption of Fig. 3. In short, the *R*_P_ factors are sensitive to peak positions because they take into account information about the intensities and approximate half-widths of the peaks. A perfect match between two spectra gives *R*_P_ = 0, while *R*_P_ = 1 means no correlation. As *R*_P_ is quite sensitive to small kinks, the rather noisy high wavenumber part of the spectrum was smoothed and is shown consistently in the following, while the low wavenumber part of the experimental spectrum was smoothed only for calculating *R*_P_ factors and is otherwise shown unsmoothed (IRMPD spectra, with and without smoothing, are shown for reference in Supplementary Fig. S4). A detailed explanation of this factor and practical examples can be found elsewhere^35^. The averaged estimated IR spectrum shows an overall good agreement in the intensity pattern, particularly for the lower wavenumber range as indicated by the low *R*_P_ value, indicating that the probabilistic PAMM clustering is able to provide adequate statistical weights for the prediction of the IR spectra.

The assumption of representing the vibrational modes of obtained conformational families by a single conformer from each cluster was further examined by assessing whether spectra within a cluster are more similar to each other than to other clusters. We calculated *R*_P_ values for all conformers from cluster **7**—selected using farthest point sampling (FPS, *cf*. SI for more information)—and all other representative conformers, and summarized them in the Supplementary Table S2. Comparison of the values in the table shows that the similarity with the representative IR signal of FPS-selected conformers within the cluster **7** is generally better than for other clusters, as the average values are higher than the value within the cluster for both low and high-wavenumber regions. The same procedure was applied to the FPS-selected conformers of all other clusters, with the similar result that the *R*_P_ were more consistent within each cluster than to others.

IR peaks in the amide I/II/III region are characteristic of the covalent bonds of the peptide backbone^44^. Normal modes in the amide III region (1200−1400 cm^−1^), which are combinations of N–H in-plane bending, C-N stretching, and C^*α*^-N bending vibrations, are complexly interrelated but are considered structurally sensitive bands for polypeptides^45^. The complexity of amide III vibrational modes makes deciphering the correlation between different secondary structures and this region of the vibrational spectrum much more difficult than for the amide I/II regions^46^. Not surprisingly, most DFT studies that proposed to relate the intensity in this range to the full range of possible backbone dihedral angles did not provide satisfactory insight into the structural sensitivity^45^. This is most likely due to the contribution of several effects, such as the dependence of the bond strengths on the backbone dihedral angles, the role of coupling between the amide III and C^*α*^-H vibrations, and, most probably, the effect of intramolecular H-bonding. Peak positions in the amide I/II regions (~1500−1700 cm^−1^) are sensitive to various secondary structures but considered only weakly affected by side-chain conformations^24,47^. However, this region is particularly interesting when solvent comes into play as it is influenced strongly by it. Many studies have hence investigated this particular region and developed frequency maps that correct the peaks for solvent effects^3–7^. To establish a link to these frequency maps and illustrate the effects of specific H-bonding in the representative conformers on the IR band of amide I, we summarize the peak shift along with the specific H-bonding pattern of the representative conformers to the experimental spectra in Table S2. Interestingly, the most favorable clusters show the smallest peak shift. Moreover, a strong coupling between ${{{{{{{{{\rm{NH}}}}}}}}}_{3}}^{+}$ scissoring modes and C=O stretches in this region has been reported^48^. Apart from this, it should be noted that the use of more than one conformation for the prediction of IR spectra of peptides in the amide I/II regions leads to better agreement with experiments, as shown previously^24,49^. However, given the overlap of bands in this region due to the many different interactions between C=O and ${{{{{{{{{\rm{NH}}}}}}}}}_{3}}^{+}$ of the different conformers, direct mixing of these signals in the amide I/II regions was not yet possible because the relative weights of the conformers were not previously known. In contrast, the excellent agreement of these regions with the experimental IRMPD spectrum in Fig. 3 elucidates these complex interactions by incorporating the many different contributions of the conformations of the protonated LeuEnk through a thorough exploration of the potential energy surface.

Peaks in the higher wavenumber range between 2700 and 3600 cm^−1^ are often influenced by a more dynamic H-bonding and are inherently more complex to interpret due to the anharmonicity. However, the agreement of the intensities in the 2900−3100 cm^−1^ range shows that an appropriate weighting of the different contributions via the PAMM conformer clusters, implicitly taking into account the H-bond statistics, is able to reproduce partly this normally highly red-shifted range after applying the standard scaling factor^50^. Particularly, it has been reported by Burke et al.^29^ that vibration modes from N–H of ${{{{{{{{{\rm{NH}}}}}}}}}_{3}}^{+}$ interacting with phenol rings of the side chains or C=O are broadened, although the extent of their shift has been underestimated by DFT calculations^14,30^. The presence of a broad low-intensity band at 3100 cm^−1^ is consistent with the dynamic H-bonds observed across the PAMM clusters, where ${{{{{{{{{\rm{NH}}}}}}}}}_{3}}^{+}$ is highly likely to donate two hydrogen atoms, indicating that ${{{{{{{{{\rm{NH}}}}}}}}}_{3}}^{+}$ (and the other amides) interact strongly with electron-rich regions of the peptide.

**Fig. 4:**
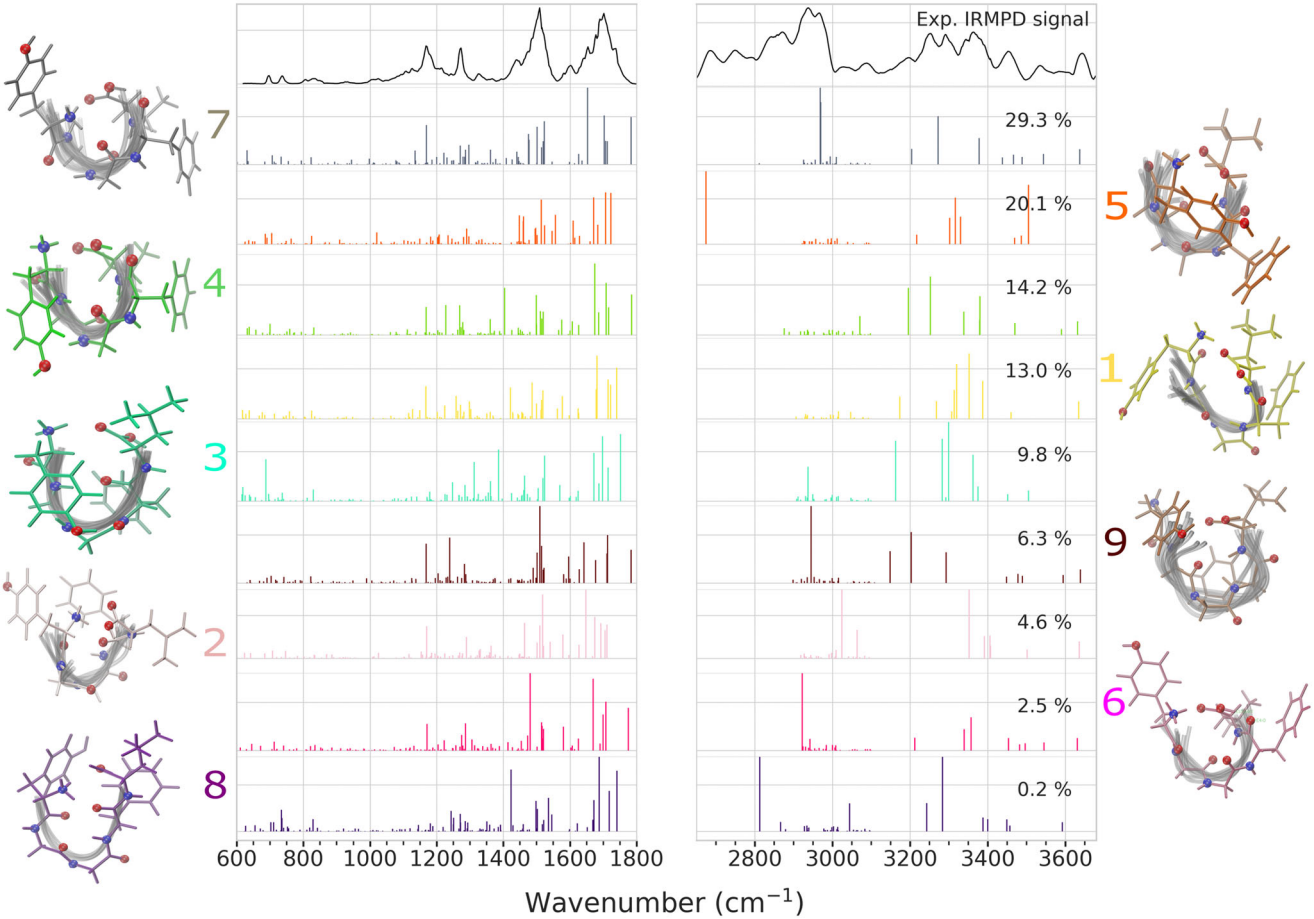
IR spectra of all PAMM conformer clusters and IRMPD spectrum. Spectra are colored according to the assigned representative conformer of each cluster, which is shown next to the corresponding spectrum. Associated weights of each conformer cluster are shown in each panel.

Closer examination of the contributions of individual conformations in Fig. 4 shows that the calculated IR spectra of the **4**, **5**, and **7** clusters agree well with the IRMPD result, indicating that the higher-weight motifs are the most abundant conformers in the experiment. The peak in the amide A region at 3643 cm^−1^ is commonly assigned to phenol O–H stretches of tyrosine, which is well reflected in the averaged spectra in terms of peak position and intensity. The phenol O–H vibrational mode was previously reported to be rather insensitive to conformational variations in LeuEnk^31^, which is confirmed when looking at individual contributions of conformers as shown in Fig. 4. Surprisingly, however, there the clusters **3** and **5** actually have no signature of this peak around 3643 cm^−1^ due to the involvement of the phenol O–H in H-bonding.

The tendency of involving COO-H and the phenylalanine carbonyl in H-bonding, visible in almost all conformers of Fig. 4 with the exception of clusters **2**, **3**, and **9**, is represented by a strongly shifted peak at 3012 cm^−1^ from the “free” position at 3584 cm^−1^ (refs. ^29,51^). Near the N–H stretch region, the shoulder-like feature between 3400 and 3500 cm^−1^, which has not been observed for single conformation spectra, could be related to the presence of weak and slightly longer NH⋯C=O H-bonds or free NH stretches (excluding ${{{{{{{{{\rm{NH}}}}}}}}}_{3}}^{+}$), which shift to higher wavenumbers and are less abundant in the peptide and consequently not as prominent^51,52^. The broadening and nature of the multiple peaks between 3200 and 3400 cm^−1^ suggest that it is unlikely to assign a single conformation with a specific NH⋯O=C bonding to this feature. However, the here presented conformational weighting sheds light on this particular region in terms of the intensity pattern and shape of the peaks.

### Hierarchical infrared spectroscopy prediction

**Fig. 5:**
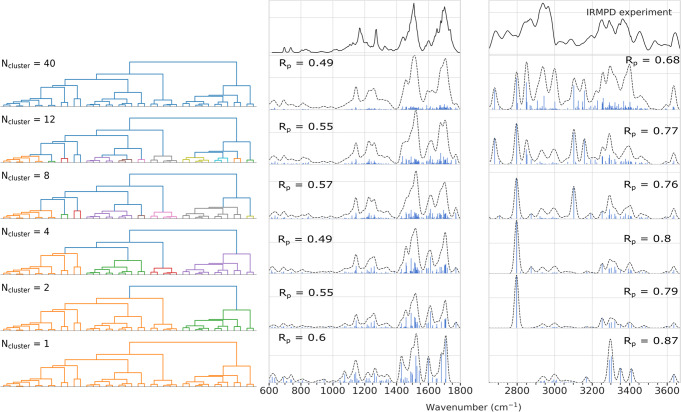
Average IR spectra for different number of merged PAMM clusters compared to the experimental IRMPD spectra of LeuEnk. Spectra are ordered by the number of PAMM clusters used and merged hierarchically as indicated by the dendrograms in the left panel. The top panel shows the spectrum considering all conformer clusters, while the bottom shows the spectrum of a single merged conformer cluster. Reliability factors *R*_P_ are reported individually for both amide A/B (middle) and amide I/II/III/V (right) regions.

Hierarchical clustering was used as illustrated by the dendrograms in Fig. 5 to mix similar conformer clusters based on their adjacency and Ward’s linkage criterion^53^. The conformers with the lowest energy of the resulting macroclusters were again selected as representative conformers for each cluster, and ab initio IR spectra were calculated for them as before. Since PAMM searches for peaks in the probability distribution using a Gaussian smearing (with a user-selected width to obtain a localized version of the Silverman rule), a narrower “probe” distribution is used to increase the number of peaks identified (i.e., *f*_points_ = 0.008 and, additionally, a quick-shift cutoff scaling of *α* = 0.9). Again, the lower wavenumber range between 600 and 1800 cm^−1^ shows good agreement according to the *R*_P_ factors. Upon closer inspection, the peak positions of the amide I and II regions agree better with the IRMPD spectrum when more conformer contributions are included. It should be noted, however, that the Pendry reliability factors here serve more as a quantitative comparison to show improvements and differences between the various obtained spectra, and their absolute values should not be overrated, as they are highly dependent on smoothing. The relatively broadband at 1630 cm^−1^, corresponding to the umbrella vibrations of the ${{{{{{{{{\rm{NH}}}}}}}}}_{3}}^{+}$ group, shows improved similarity with the experimental signal in terms of intensity pattern and broadening when more conformers are included. Inclusion of more conformers also improves the amide II region and leads to a more similar shoulder as observed in the experiment at wavenumbers below 1500 cm^−1^. This band could be attributed to a *σ*-NH scissoring mode, sensitive to the orientation of the H-bonds in which its found^47^.

In the amide A region, the phenol OH stretching mode remains unchanged, further demonstrating the insensitivity of this band to conformational changes. On the other hand, the amide A/B regions, which include localized CH and NH stretching vibrations, improve dramatically as more conformations are included in the averaged IR spectrum. High-frequency CH and NH vibrational modes of molecules are often coupled with degenerate overtone and combination bands, as the vibrational configuration interaction causes intermixing of the high-frequency stretching states.

To support the argument that a single representative conformer from each cluster represents the contribution of the whole cluster to the complete spectra, a comparison of the IR signal of 10 randomly selected conformers within cluster **7** is given in Supplementary Fig. S6. After geometry optimization, these conformers are generally pushed to some local minima within the cluster, which could be identified by finer probing of the conformational phase space, i.e., by tuning *f*_points_, with PAMM. Thus, differences are expected in the calculated IR spectra from the individual conformers within each cluster (cf. Supplementary Fig. S7). Although the low-frequency region does not seem to be affected as much as the high-frequency region, as shown by the averaging of the spectra in Supplementary Fig. S8, suggesting greater structural similarity associated with this wavenumber range. Most importantly is that the representative structure is the energetically preferred structure even after geometry optimization (cf. Supplementary Table S1). Signals from other conformers in this cluster will thus most likely not contribute to the same extent as the representative conformer. Related to this, increasing the number of clusters to a still reasonable number seems to improve the IR prediction as more local minima and their associated IR signals are considered. Keeping this in mind, it is in general quite difficult to assign specific conformers to IR signals, especially when multiple conformers with different H-bonding patterns are present in the experiment but not in the conformational ensemble (cf. high-frequency part of the IR spectrum in Supplementary Fig. S7). However, further breakdown of the cluster facilitates the interpretation of this region in terms of intensity patterns and peak positions. Nevertheless, one must carefully consider when to stop adding conformations, as this may result in adding conformations that are incorrectly weighted if too large a number of clusters are identified and clusters are incorrectly populated due to missing samples.

## Conclusions

At finite temperatures, multiple peptide conformers separated by low-energy barriers and affected by different intramolecular H-bonding are present in a macroscopic ensemble and hence contribute to the experimental observations^2,21,54,55^. In this work, the importance of conformational ensembles for a robust IR prediction was investigated which resulted in a good qualitative agreement with the experimental IRMPD spectrum for protonated LeuEnk in the gas phase. In particular, we combined unsupervised machine learning with replica-exchange molecular dynamics (REMD) simulations and ab initio calculations to incorporate a realistic representation of a canonical ensemble in the spectra prediction. An important part of the study of conformations generated by free-energy methods such as REMD is the partitioning of the conformational space using an agnostic analysis approach to avoid potential biases that could lead to contrived results and thus misleading conclusions^36,56^. As a possible solution to this, we have shown how a PAMM clustering analysis applied to a calculated collective variable space of SOAP kernels is able to categorize the conformational ensemble into different recurrent molecular motifs based on a kernel density estimation. Furthermore, it is shown that investigating the H-bonding dynamics, as obtained by a PAMM analysis, shed further light on specific H-bonding influences on the secondary structure motifs of LeuEnk.

Native-like conformations that are stable in solution can turn metastable for biomolecules studied in the gas-phase experiments, due to the intact H-bonding network and the enhanced electrostatic interactions, leading to rather unusual transitions pathways between conformations^57,58^. Thus, the experimental preparation is crucial, as it can affect the final outcome of the spectroscopic experiment, e.g., through kinetic trapping^41^. To address this better, we decided to repeat previous IRMPD spectroscopy experiments with LeuEnk, with particular attention to the preparations. In contrast to previous experimental and theoretical studies of LeuEnk^29,32^, the current preparation leads to a very good agreement with our predicted IRMPD spectrum, which is probably related to a better agreement between the two ensembles in the theoretical and experimental approach. In conclusion, considering only a few lowest energy conformers and neglecting the relative importance of the conformers, as it is often common practice in simulation approaches, leads to an inadequate description of the conformational space explored by LeuEnk in gas-phase experiments and, consequently, to a misleading interpretation of IR spectroscopy. We corroborate that averaging the calculated IR spectra of representative motifs, based on the weighting factor obtained from the relative population of a PAMM cluster, can be used as a reasonable approach for representing the general structural canonical ensemble. Specifically, the comparison between the averaged IR and the IRMPD spectra illustrates that the averaged spectrum reproduces well the intensity pattern and the main peak positions, capturing the main features of the vibrational spectra although some particular features may be missing due to the specific way a representative conformer is chosen here. Especially in the high-wavenumber region that is strongly influenced by H-bonding, we conclude that the existence of multiple low-energy conformers should not be neglected. For a further evaluation of the importance of the conformational ensemble, we performed thus a hierarchical clustering based on the information of a PAMM analysis to evaluate the effect of breaking down “macroclusters” on the averaged IR spectra. The improvements observed in the Pendry reliability factor *R*_P_ for amide A/B and amide I/II/III regions indicate that the importance of unraveling non-Gaussian features in the potential energy surface to explain the IR fingerprints are strongly correlated to changes in the backbone conformation and H-bonding patterns. However, the stabilizing role of the ${{{{{{{{{\rm{NH}}}}}}}}}_{3}}^{+}$ group in H-bonding networks defines a cutoff in the number of conformations contributing to the experimental IR spectra due to the appearing discrepancies in peak positions and intensity patterns in amide A/B regions.

As final remarks, we expect that performing more sophisticated ab initio approaches such as dynamic IR absorption with ab initio molecular dynamics^22^ in combination with our approach could further improve the prediction as local anharmonic contributions from each representative conformer basin are currently neglected. Additionally, the IR prediction could benefit from including nuclear quantum effects^59,60^. Nevertheless, we speculate that some anharmonic effects are implicitly accounted for in our approach, since we are partially sensitive to them by sampling the conformational space with REMD. In summary, the approach presented here provides a unique opportunity to broadly explore the conformational space and to include these invaluable information in explaining the spectroscopic fingerprints of large biomolecular systems based on a compromise between rather low calculation expenses of REMD simulation and expensive ab initio calculations.

## Methods

To efficiently capture the potential energy surface (PES) of LeuEnk, replica-exchange molecular dynamics (REMD) simulations based on the Amber ff14SB^61^ force field have been performed. The analysis of the REMD trajectory at 300 K allows evaluation of the unbiased canonical probability distribution of physical quantities at that temperature, while replicas simulated at higher temperatures and their replica exchange using the Metropolis-Hastings algorithm ensure that phase space is adequately explored^62^. We used the Smooth Overlap of Atomic Positions^63–66^ (SOAP) feature space as a descriptor to unambiguously represent structural information about conformers found in the REMD trajectory at 300 K. The resulting SOAP kernels are generic descriptors of local structures that discretize the three-body correlation functions around each atom and capture its relationship to neighboring atoms. SOAP kernels are typically used to establish relationships between different local atomic environments of other molecules and to calculate molecular similarity^64,67^. We then applied the Probabilistic Analysis of Molecular Motifs (PAMM) technique on this data to identify recurrent molecular conformations of various stable and metastable states observed during the REMD. Detailed explanations of the experimental and simulated methods can be found in the supplementary information.

## Supplementary information

Supplementary Information

Description of Additional Supplementary Files

Supplementary Data

## Data Availability

Additional information regarding the experimental and simulation methods, dimentionality reduction of SOAP kernels, IRMPD spectrum before smoothing, 3D-structures of representative LeuEnk motifs, and a comparison of IR signals of FPS-selected conformers within a cluster are available in supplementary information. Cartesian coordinates of DFT geometry optimized conformers of all clusters as well as the initial and final conformers of REMD simulation are provided in Supplementary Data.
