# Supplementary material for: Reconstructing the infrared spectrum of a peptide from representative conformers of the full canonical ensemble
Source: Commun Chem. 2023 Mar 3;6:46. doi: 10.1038/s42004-023-00835-3 (PMC9984374; doi:10.1038/s42004-023-00835-3)
Supplement: Supplementary file 1 — Supplementary Information [file 42004_2023_835_MOESM1_ESM.pdf]

Supporting Information: Reconstructing the infrared spectrum of a peptide from representative conformers of the full canonical ensemble

Amir Kotobi,[†] Lucas Schwob,[*,†] Gregor B. Vonbun-Feldbauer,[‡] Mariana Rossi,[¶] Piero Gasparotto,[§] Christian Feiler,[‖] Giel Berden,[⊥] Jos Oomens,[⊥] Bart Oostenrijk,[#,†] Debora Scuderi,[@] Sadia Bari,[*,†,△,#] and Robert H. Meißner[*,∇,‖]

†*Deutsches Elektronen-Synchrotron DESY, Hamburg, Germany*

‡*Hamburg University of Technology, Institute of Advanced Ceramics, Hamburg, Germany*

¶*Max Planck Institute for the Structure and Dynamics of Matter, Hamburg, Germany*

§*Scientific Computing Division, Paul Scherrer Institute, Villigen, Switzerland*

‖*Helmholtz-Zentrum Hereon, Institute of Surface Science, Geesthacht, Germany*

⊥*Radboud University, Institute for Molecules and Materials, FELIX Laboratory, Nijmegen, Netherlands*

#*The Hamburg Centre for Ultrafast Imaging, Hamburg, Germany*

@*Institut de Chimie Physique, CNRS, Université Paris-Saclay, Orsay, France*

△*Zernike Institute for Advanced Materials, University of Groningen, Groningen, Netherlands*

∇*Hamburg University of Technology, Institute of Polymers and Composites, Hamburg, Germany*

E-mail: lucas.schwob@desy.de; sadia.bari@desy.de; robert.meissner@tuhh.de

Supplementary Methods

Simulation Details

Molecular dynamics (MD) simulations combined with empirical force fields is a frequently used tool to obtain long and ergodic trajectories of biomolecules in the gas phase. The ability to assess the metastable states and the resulting free-energy landscapes of proteins thus became a valuable aid in the interpretation of experimental data.[S1–S3] In our case, simulations of an N-terminal protonated LeuEnk ($[\text{YGGFL+H}]^+$) (cf. Supplementary Figure S1) in the canonical ensemble (NVT) were run for 200 ns. The leap-frog MD algorithm[S4,S5] was used for the simulations in the gas phase with a time step of 0.5 fs with no cutoffs for non-bonded interactions and no periodic boundary conditions applied. In addition, we performed Replica Exchange Molecular Dynamics (REMD) simulations on the basis of the Amber ff14SB force field[S6] in GROMACS version 2018.8 to efficiently sample the potential energy surface (PES). Exponentially distributed[S7] REMD simulations were carried out at temperatures of 300, 352, 413, 481, 559, and 648 K. Temperature coupling during the simulation was realized with the velocity rescaling temperature control of Bussi et al.[S8] and in absence of any bonded constraints, i.e. no SHAKE or RATTLE. Infrared (IR) spectra have been subsequently obtained by first tightly geometry optimizing relevant conformers until energy changes became smaller than 10^{-8} a.u. and 5^{-5} a.u. for the electronic and atomic geometry optimization, respectively, using the ORCA electronic structure package[S9] with BP86/def2-TZVP[S10–S12] level

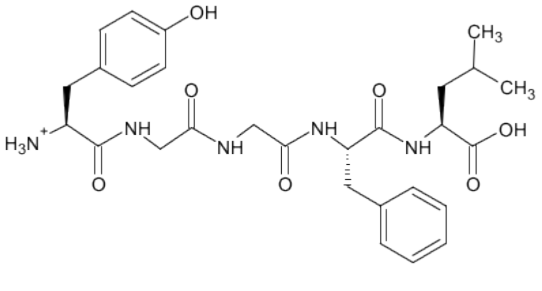

Supplementary Figure S1: 2D structure representation of N-terminus protonated LeuEnk

of theory and Grimme's dispersion correction D3.[S13] Subsequent to geometry optimization, harmonic frequency calculations were performed for amide A/B ($\sim 2700 - 3700\,\mathrm{cm}^{-1}$) down to amide I/II/III/V ($\sim 600 - 1800\,\mathrm{cm}^{-1}$) vibrational modes with the same level of theory. Amide A/B stretching modes were scaled with a value of 0.989 on the basis of previously published scaling values[S14] in order to compensate the lack of anharmonicity.

Atomic Descriptors

Unfortunately, the amount of data generated in MD simulations often hampers the analysis and visualization, and ultimately the understanding, of complex processes. As a consequence, a more compact representation of such data is required to effectively describe the kinetics of complex conformational changes between different states of a protein. For this reason, atomistic trajectories are usually represented by selecting a small set of collective variables (CVs) (or descriptors) that are functions of the atomic coordinates and which provide a satisfactory description of the thermodynamic and kinetic properties of the system while having a low dimensionality.[S15]

Several global representations of atomic coordinates have been suggested to analyze large atomistic datasets by incorporating physical invariances (i.e. rotations, translations and permutations of identical atoms), simplifying the description of complex systems.[S16–S19] However, determining important structural features characteristic of metastable states is far from trivial due to the highly dynamic exploration of the free-energy landscape. Moreover, identifying significant motifs is a tedious task given the often vast amount of simulation data.

Unsupervised machine learning (ML), e.g. clustering and dimensionality reduction methods, have proven very effective in disentangling the complexity of high-dimensional structural manifolds of biomolecules into structure-property relationship maps.[S20–S24] Thus, it is necessary to convert the atomic coordinates of the trajectory frames from the REMD simulation into some set of useful CVs to calculate relevant statistical quantities.[S25] Here, we used the Smooth Overlap of Atomic Positions[S26–S29] (SOAP) approach. SOAP kernels are generic de-

scriptors of local structures which discretize three-body correlation functions centered around each atom, capturing its relationship with neighboring atoms, and the relationships between sets of neighbors.[S27,S30] Moreover, SOAP kernels are designed to capture physical symmetries such as invariances to translations, rotations and permutations of atoms.[S22,S31]

Within the SOAP formalism, a local atomic environment ρ_i^α of an atom i of chemical species α is described by a set of atomic densities, represented by Gaussians centered on neighboring atoms j,

$$\rho_i^\alpha(\mathbf{r}) = \sum_j \exp(-\frac{|\mathbf{r}-\mathbf{r}_{ij}|^2}{2\sigma^2}) f_\mathrm{c}(|\mathbf{r}_{ij}|) \tag{1}$$

where f_c is a cutoff function that selects smoothly atoms from a local environment within a radius r_c, chosen here to be 6 Å, from the central atom. The width of the Gaussians is set here to $\sigma = 0.4$ Å. To prevent the number of local environments becoming prohibitively large for further calculations, SOAP environments were created only for the nitrogen atoms in the molecular backbone. It should be noted, though, that these environments contain all the atomic contributions of other chemical species within the cutoff. A kernel between local environments of two LeuEnk conformations, denoted A_i and B_j, is usually formed via a rotationally-averaged squared overlap kernel[S32] of the smooth atomic densities,

$$k(\mathrm{A}_i, \mathrm{B}_j) = \int_{\mathrm{SO(3)}} \left| \sum_\alpha \int_{\mathbb{R}^3} \rho_{\mathrm{A}_i}^\alpha(\mathbf{r}) \rho_{\mathrm{B}_j}^\alpha(\mathbf{r}) \mathrm{d}\mathbf{r} \right| \mathrm{d}\hat{R} \tag{2}$$

The advantage of SOAP environments is that this integral is analytically solvable with spherical harmonics, controlled by l_max the maximum angular degree of the spherical harmonics, and orthogonal radial basis functions, controlled by n_max the maximum number of the radial basis functions.[S26] Here, we chose standard values of $n_\mathrm{max} = l_\mathrm{max} = 6$.

Pairwise environment similarities of peptide conformations A and B are stored in a similarity matrix $C_{ij}(\mathrm{A}, \mathrm{B}) = k(\mathrm{A}_i, \mathrm{B}_j)/\sqrt{k(\mathrm{A}_i, \mathrm{A}_i) k(\mathrm{B}_j, \mathrm{B}_j)}$. In order to compare the similarity between molecular conformations of LeuEnk here an averaged structural kernel[S26] $K(\mathrm{A}, \mathrm{B}) = \frac{1}{N^2} \sum_{ij} C_{ij}(\mathrm{A}, \mathrm{B})$ is used. Having defined the kernel between the conformations,

a kernel distance[S33] for conformers A and B is obtained from

$$D(\mathrm{A},\mathrm{B}) = \sqrt{K(\mathrm{A},\mathrm{A}) + K(\mathrm{B},\mathrm{B}) - 2K(\mathrm{A},\mathrm{B})} \tag{3}$$

The kernel distance D was then used as the metric for representing and clustering the molecular conformations.

Probabilistic Analysis of Molecular Motifs (PAMM)

Since the probability density of sampling conformations in MD simulations is based on a Boltzmann distribution, where each point represents a molecular conformation in the configuration space, clustering algorithms can aid to partition the conformational ensemble into distinct subensembles according to the (usually high-dimensional) distances between points. Partitioning the free-energy landscape into groups of metastable basins, and demonstrating the connection between them, is particularly useful to reproduce qualitatively the system's dynamical behaviour and to identify the transformation pathways between different states.[S34–S38]

Here, PAMM[S39,S40] was employed to identify recurring molecular conformations of different metastable states sampled during the REMD. PAMM is specifically designed to work in relatively high-dimensional spaces and is able to partition the underlying probability distribution function into modes corresponding to different conformers. Firstly, a kernel density estimation (KDE) is performed on a grid representing the data which was extracted by choosing a small set of landmarks from the full data set by using a farthest point sampling (FPS) algorithm.[S41] FPS attempts to span the conformation space homogeneously by selecting landmark molecular conformers $\{\mathrm{X}_i\}$, where $i = 1, \ldots, N$ and N is the total number of landmarks to select, using the kernel distance D between LeuEnk conformations from the

full set of conformations $\mathcal{Z}$ according to the criteria

$$X_{j+1} = \arg\max_{Z \in \mathcal{Z}} \left[\min_{i \leq j} \big(D(X_i, Z) \big) \right] \tag{4}$$

in which the first conformer $X_1 \in \mathcal{Z}$ is selected randomly. An adaptive multivariate kernel bandwidth for the KDE on the landmark points results then from a localized version of Silverman's rule defined by the smoothing parameter $f_{\text{points}} = 0.05$.[S40] This parameter can be used to optimize the number of identified clusters if done with due care, as it sets the automatically selected bandwidth for kernel density estimation either by a fraction of the total number of points f_{points} or by a fraction of the variance of the entire dataset f_{spread}. Apart from this, a slight change in the quick-shift cutoff (usually by changing the so-called scaling factor α) can also help to divide the data into more clusters.[S39] We performed a careful parameter search to find the optimal number of grid points and Gaussian kernel widths to obtain a robust and smooth estimate of the high-dimensional probability density function underlying the data, as discussed more in detail elsewhere.[S22,S35,S40] Following this, a localized quick-shift variant[S40] is used to identify to which cluster the landmark conformers belong. PAMM produces a set of fingerprints or more specifically probability motif identifiers (PMI), which are simple Gaussian naive Bayes classifiers in the original formulation, trained to distinguish between different recurrent motifs in the simulation, while providing at the same time a probabilistic interpretation. Since often different metastable structures are sampled from different free-energy basins that are very close (hence very similar), it is convenient to merge multiple PMIs together in an hierarchical clustering approach. One can see this step as a coarse-graining of the structural landscape often employed to properly extract informative structure-property relationships, e.g. to capture the hierarchical nature of a peptide's free-energy landscape.[S35,S37,S42–S44] Briefly, identification of such macroclusters is done by bootstrapping the clustering step to determine an adjacency matrix, $\mathbf{A} \equiv (a_{ij})$, capturing the overlap between clusters. The metric used to compare two distinct PMIs i

and j during the hierarchical clustering is given by $d_{ij} = -\ln(a_{ij}/\sqrt{a_{ii}a_{jj}})$, where a_{ii} values are the diagonal elements indicating how robust the determination of the i-th cluster was, while a_{ij} are the off-diagonal terms, indicating the fuzziness of the cluster borders.

Given the high-dimensional nature of SOAP kernel distances, a PAMM analysis based on the full descriptors would be computationally infeasible and also too susceptible to insignificant structural features. Thus, a Principal Component Analysis (PCA) is applied to reduce the dimensionality of our structural descriptors. We use the cumulative variance ratio carried by an increasing number of principal components (PCs) to estimate the intrinsic dimensionality of our structural descriptors. As shown in Supplementary Figure S2, we find that in order to recover ∼92% of the variance in the data, ten principal components are sufficient. Finally, we use the first two principal components as a way to project the entire structural landscape[S22] sampled in our REMD simulations into an easily interpretable two-dimensional map shown in Figure 1a. The weighting factors w in Table 1 take into account the internal energy of the system in that specific state and include entropic effects to assess the possible presence of multiple conformations in the experiment by relating the population of a single cluster to the total population. It should be noted that the identified representative conformers were geometrically optimized based on density functional theory (DFT) before the corresponding *ab initio* IR spectra were calculated. However, we paid particular attention to the fact that the geometrically optimized conformation falls in the same PAMM cluster as before (cf. Supplementary Figure S3).

Aside from this, recurrent hydrogen bonding configurations, denoted as H-bonding in the following, are obtained following an earlier approach of Gasparotto and Ceriotti[S39]. Within this, the geometry of an H-bond is defined as the combination of three distances between the donor (D), acceptor (A) and hydrogen (H): (i) the proton-transfer coordinate $\nu = |d_{\mathrm{DH}} - d_{\mathrm{AH}}|$, (ii) the symmetric stretch coordinate $\mu = |d_{\mathrm{DH}} + d_{\mathrm{AH}}|$ and (iii) the acceptor-donor distance $r = d_{\mathrm{AD}}$. Here we focus only on the nitrogen in the amide groups as a donor and oxygen as an acceptor for an H-bond in the sense of $\mathrm{N-H\cdots O}$, since this H-bonding is mainly

responsible for the secondary structure and neglect H-bonds of the type $O - H \cdots O$. To consider only relevant H-bond configurations, a cutoff distance of 4.5 Å was considered for μ. The triplet set of (ν, μ, r) is then used to partition the data into different H-bonding clusters as shown in Figure 2. The number of H-bonds accepted by oxygen, s_A, and donated by nitrogen, s_D, given in Table 1 is calculated by using PAMM only on the subset of conformers belonging to each cluster.

Principal Component Analysis

Principal component analysis (PCA) is commonly used to estimate how many components are actually required to describe a set of data. PCA is commonly used for dimensionality reduction by projecting each data point onto only the first few principal components to obtain lower dimensional data while preserving as much of the variation in the data as possible. Accordingly, the first principal component can be defined as a direction that maximizes the variance of the projected data. Hence, we used the cumulative explained variance ratio as a function of the number of components in order to reduce the dimensionality of the Smooth Overlap of Atomic Positions (SOAP) kernels. Consequently, Supplementary Figure S2 shows the cumulative explained variance versus the first 40 principal components.

Low-dimensional Embedding of *ab initio* Conformers

The conformation with the lowest energy of each identified PAMM conformer cluster was selected, and after confirming that they belonged to the same PAMM cluster after geometry optimization with DFT, its SOAP distance was recalculated, as described in more detail in the main text. For reference, the geometry-optimized conformers were embedded in the two-dimensional map shown in Supplementary Figure S3.

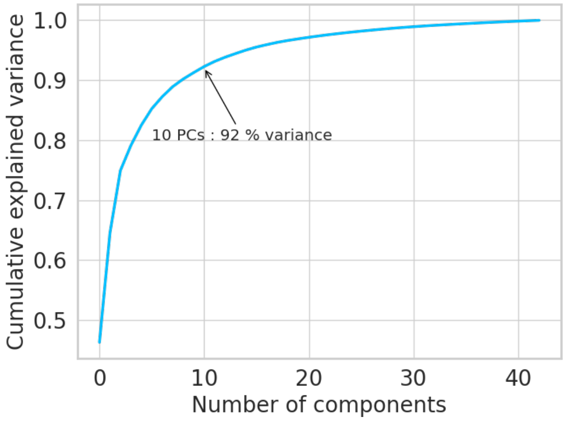

Supplementary Figure S2: Relationship of the cumulative explained variance of the SOAP kernels to the number of principal components (eigenvectors) of the PCA. The cumulative explained variance is a statistical measure of how much information, i.e. variation, can be retained in the data set as more and more principal components are included.

Experimental Section

Room temperature experiments in the fingerprint region, $600 - 1800\,\mathrm{cm}^{-1}$, were performed at the Free Electron Laser for Infrared eXperiments (FELIX) laboratory in Nijmegen (The Netherlands). The Free-Electron Laser (FEL) was coupled to a Bruker AmaZon ETD quadrupole ion trap mass spectrometer which was modified to have optical access to the ion trapping region as is described in detail elsewhere.[S45] The IR frequency was calibrated using a grating spectrometer. Protonated LeuEnk ions were generated from solution by ESI, mass-to-charge isolated in the trapping region, and irradiated with a single infrared laser pulse from the FEL (5-100 mJ per pulse, bandwidth 0.4% of the IR frequency) to induce wavelength-dependent IRMPD. Precursor and fragment ion intensities were determined from six averaged mass spectra at each IR frequency.

The IRMPD spectra in the $2700 - 3700\,\mathrm{cm}^{-1}$ range have been recorded at the Centre Laser IR d'Orsay (CLIO) FEL facility in Orsay (France)[S46] using a modified 7 T hybrid FT-ICR mass spectrometer (APEX-Qe Bruker) coupled to the table-top optical parametric oscillator/amplifier (OPO/OPA, Laser Vision). This experimental setup has been previously described in detail elsewhere[S46] and for the first time here implemented with a 10 Hz Nd:YAG

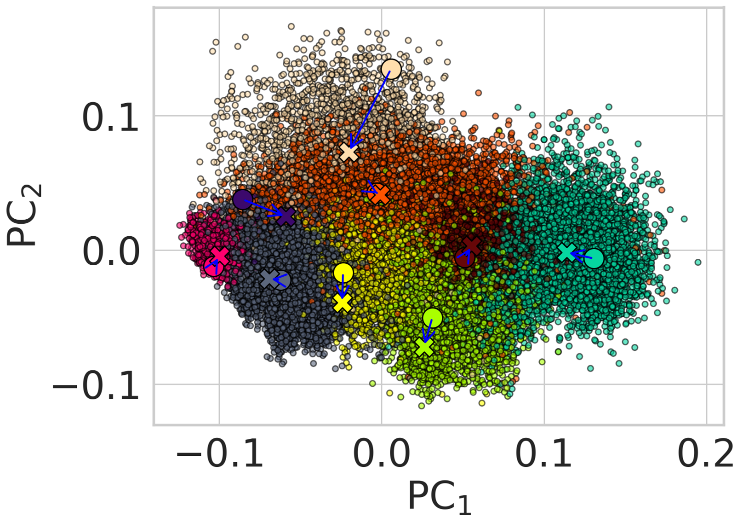

Supplementary Figure S3: 2D-PCA representation of LeuEnk conformers from a REMD simulation along with an embedding of DFT geometry optimized conformers of each identified representative PAMM conformer. Circles and crosses indicate the position of the original PAMM conformer and the geometry optimized conformers in the 2D PCA map, respectively.

pumped OPO/ OPA system (Surelite II,10-Continuum, Laser Vision). Briefly, protonated LeuEnk ions were delivered from the solution to the gas phase by ESI with a typical extraction voltage of 4 kV and desolvation temperature of $150 - 200\,^{\circ}$C. Ions were accumulated, pre-mass-selected and thermalized in a quadrupole-hexapole interface and then pulse extracted towards the FT-ICR cell maintained under high vacuum ($< 10^{-9}$ mbar) and at room temperature. Mass-selected ions were stored and irradiated for 2 s with the OPO/OPA light in the frequency range of interest. In order to increase fragmentation, ions were also exposed to an auxiliary broad-band CO_2 laser synchronized with the OPO/OPA (Universal Laser System, 10 W, continuous wave operation centered at $\lambda = 10.6\,\mu$m), at the beginning of the OPO/OPA irradiation period and for times varying between 7 to 12 ms. The CO_2 laser pulse length was adjusted to avoid photo-dissociation of the molecule by the CO_2 laser alone while promoting the fragmentation in the presence of the OPO/OPA. The IR spectra in both ranges are obtained by plotting the IRMPD yield as a function of IR frequency, where the yield is defined as $-\ln\left(I_\mathrm{p}/\sum\left(I_\mathrm{f}+I_\mathrm{p}\right)\right)$ with the precursor and fragment ion intensities (I_p and I_f). In the 600 to 1800 cm^{-1} fingerprint region, the IR yield was further linearly

corrected for the frequency dependent variation of the FEL pulse energy.[S47]

IR Multiphoton Dissociation (IRMPD) Spectroscopy

The complete IRMPD spectrum was smoothed throughout the main manuscript text in order to obtain robust Pendry reliability factors R_{P} for comparing the theoretical and experimental IR spectra. The smoothed and raw IRMPD spectrum are given in Supplementary Figure S4 for reference.

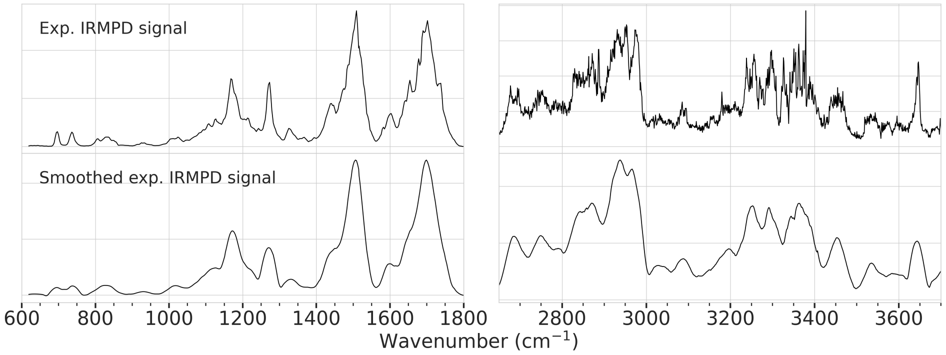

Supplementary Figure S4: Raw data versus the smoothed IRMPD experimental spectrum used for comparison to predicted IR signals of a protonated LeuEnk peptide at room temperature.

Representative 3D-structures of LeuEnk Motifs

IR Signal Comparison within a Cluster

Farthest Point Sampling (FPS) was applied to the distance kernel D to achieve a homogeneous exploration and sampling of the high-dimensional phase space. The 10 conformers indicated by large dots in Supplementary Figure S6 were randomly selected from the **7** cluster in this way. To calculate vibration frequencies using *ab initio* methods, the conformers selected by FPS had to be geometrically optimized, which are indicated by the crosses in Supplementary Figure S6. Supplementary Table S1 summarizes the changes in pairwise kernel distances as well as the relative DFT energies of the FPS conformers to the representative

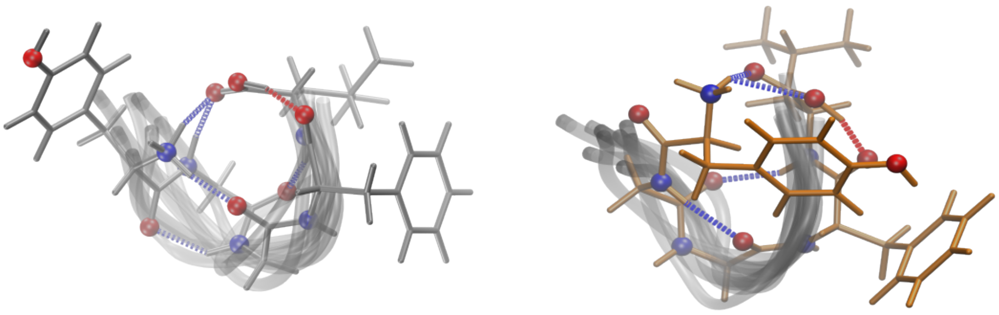

Supplementary Figure S5: 3D-representation of the two clusters (**5** and **7**) with the highest weight, given in percent. Oxygen and nitrogen atoms are highlighted with blue and red colors, respectively. Hydrogen bonds formed between NH···O and OH···O are indicated by corresponding colored dotted lines.

conformer. Taking into account the initial position of the conformers, the position of the

Supplementary Table S1: Kernel distances (cf. eq. 3) of FPS-selected conformers to the representative conformer of cluster **7** before, denoted by D^{REMD}, and after *ab inito* geometry optimization, denoted by D^{DFT}. The relative *ab initio* energy difference between the representative conformer and FPS selected conformers is given in the last column.

Conformer	D^{REMD}	D^{DFT}	ΔE^{DFT} / eV
1	0.0052	0.0018	0.46
2	0.0089	0.0043	0.38
3	0.0053	0.0067	0.68
4	0.0114	0.0077	0.24
5	0.0159	0.0078	0.57
6	0.0113	0.0109	0.16
7	0.0075	0.0114	0.16
8	0.0058	0.0123	0.40
9	0.0192	0.0155	0.10
10	0.0101	0.0162	0.13

geometrically optimized conformers illustrates the highly complex transition pathways between conformations and the complex and shallow landscape of conformational free energies. In summary, random selection with FPS usually does not lead to the global minimum of a representative cluster. However, selection via the conformer with the lowest energy from the REMD energies seems to work very well here, since the resulting *ab initio* energy of the representative conformer is still lower than all geometry-optimized FPS-selected conformers.

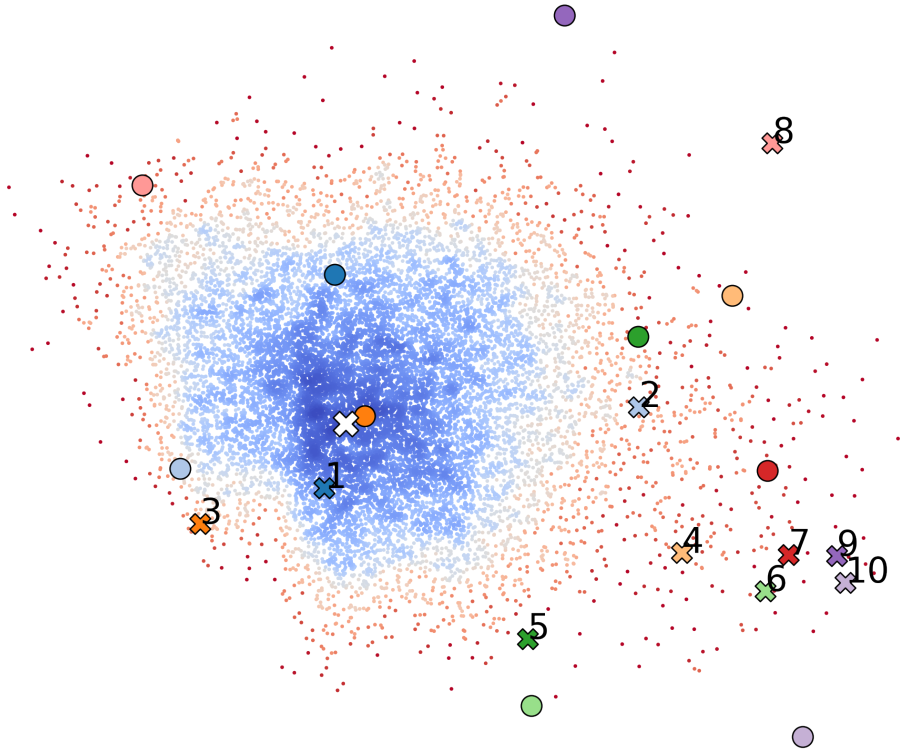

Supplementary Figure S6: Scatter plot of cluster **7**. Points are colored according to the logarithm of the KDE determined from PAMM, which illustrates the free energy landscape (blue: low free energy; red: high free energy). FPS selected conformers, before and after *ab inito* geometry optimization, are indicated with circles and crosses, respectively. The white cross indicates the position of the representative conformer.

The resulting IR spectra of the FPS-selected conformers, ordered by their pairwise distances from the representative conformer of cluster **7**, are shown in Supplementary Figure S7. The distinct peak position and shape of the amide I band in Supplementary Figure S7 is an indicator that all conformers possess a similar backbone conformation. The σ-NH-scissoring mode at $1500\,\mathrm{cm}^{-1}$,[S48] which is sensitive to the angle between the donor and acceptor of the H-bond, appears in all FPS-selected conformer spectra, but in different shapes and intensities, indicating, however, some backbone flexibility. Controversially, the IR peaks in the amide A/B regions of FPS-selected conformers are highly variable and probably strongly influenced by dynamic H-bonding, which is observed even in the same cluster. This becomes clear when looking at the main peaks of the N−H and C−H stretching modes between 3000 and 3300 cm^{-1}, which are more or less visible in all IR spectra of the FPS-selected conformers, but vary greatly in intensity and shape.

In addition, FPS was used to select 10 conformers from each of the clusters. For each conformer, the *ab initio* IR spectrum was calculated, and the resulting (unweighted) average spectrum is shown in Supplementary Figure S8. It should be noted that the geometry optimization of the conformers performs a kind of implicit, and physical, weighting, as the 10 conformers selected with FPS are shifted to their nearest local minimum. The bands in the low-frequency region are relatively consistent with each other, although differences are observed in the low-frequency region of the amide bands, suggesting different backbone configurations between the clusters – as a comparison with the conformers shown in Figure 1a of the main manuscript would suggest. Changes in amide I/II regions (1200 - 1750 cm^{-1}) in the averaged IR spectra of Supplementary Figure S8 could serve as a marker for conformational changes when comparing the signals to different conformers shown in Figure 1a. In addition, the variations in the high-frequency region are quite large, which can be attributed to the larger hydrogen bonding dynamics of the peptide motifs in this region.

Supplementary Table S2 summarizes the R_P values of IR spectra for all FPS-selected conformers from cluster **7** and all other representative conformers.

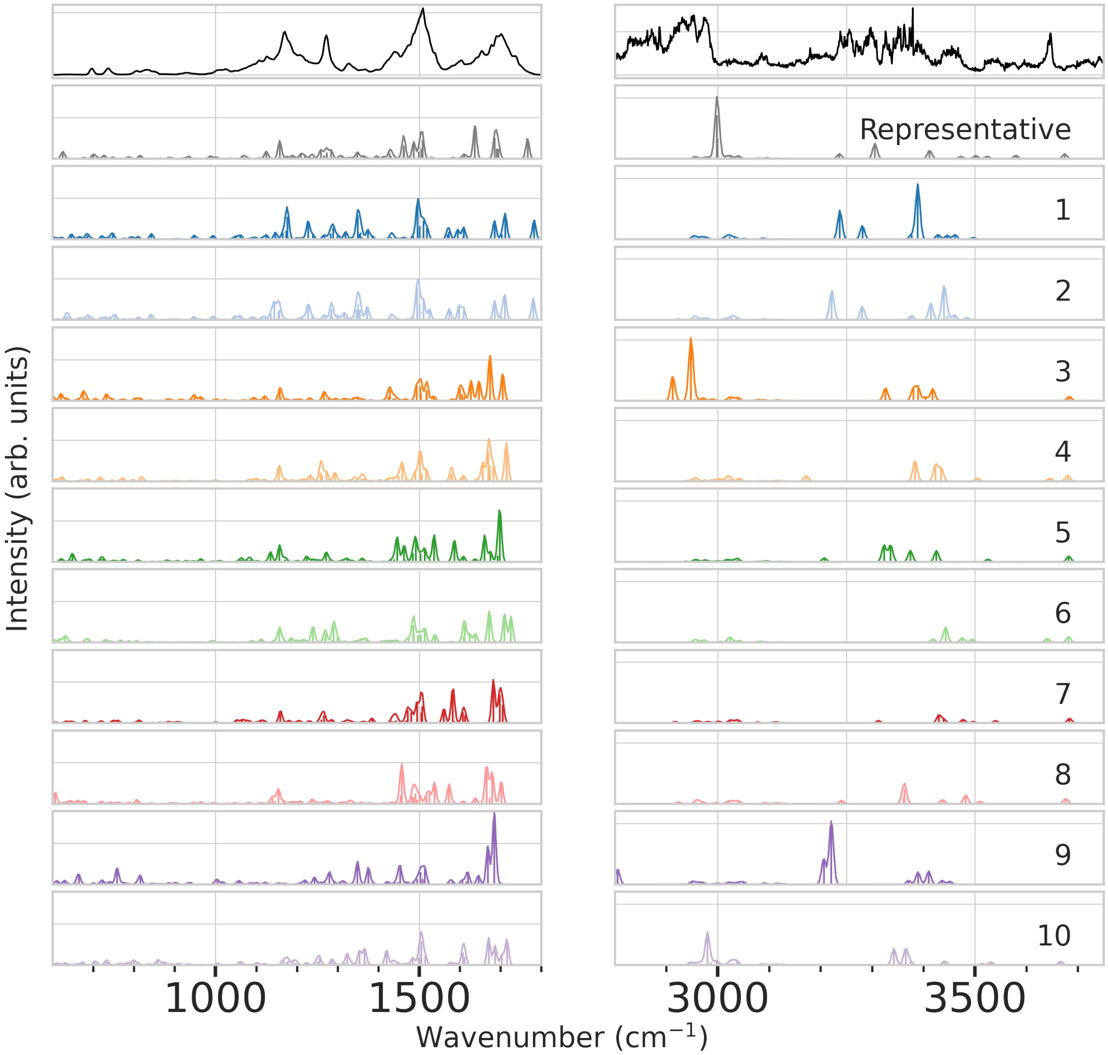

Supplementary Figure S7: IR spectra of the 10 FPS-selected conformers from cluster **7**.

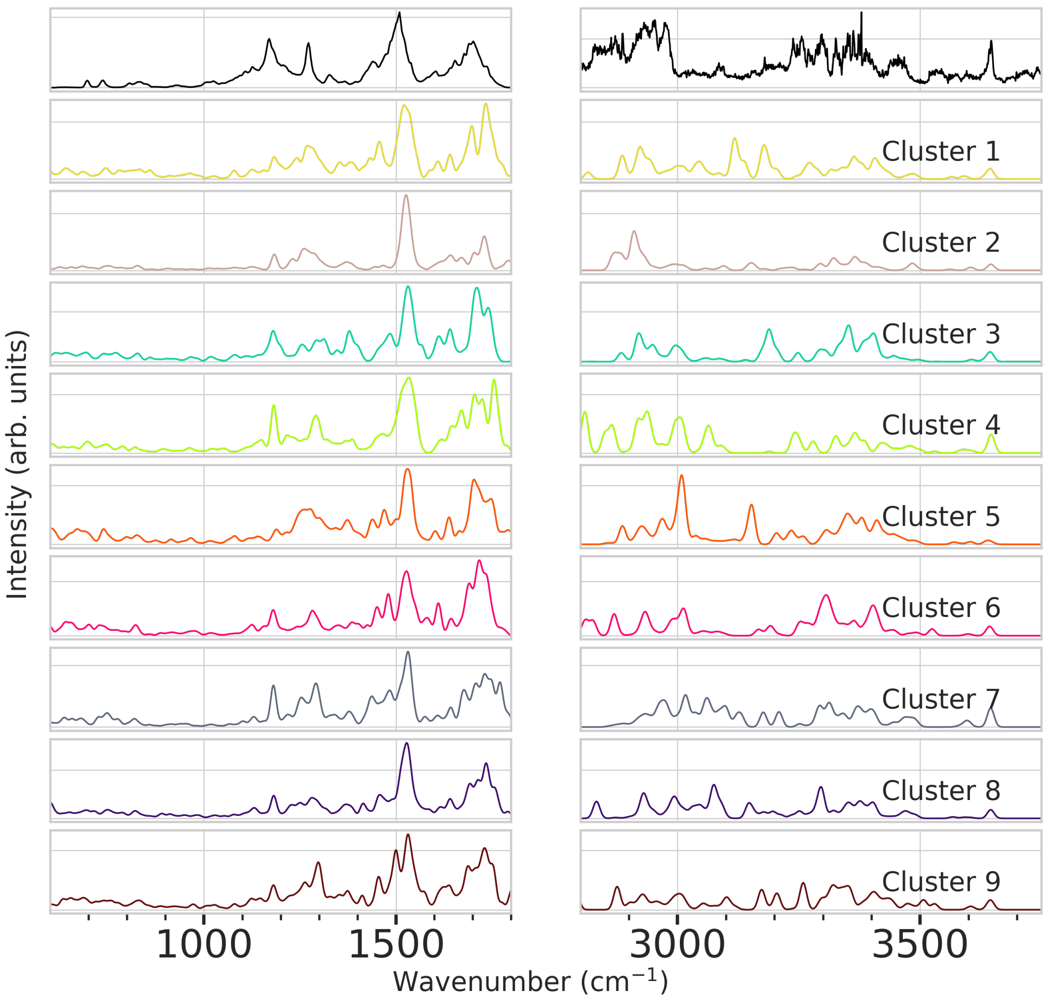

Supplementary Figure S8: Averaged IR spectra for each cluster calculated from 10 conformers selected via FPS from all REMD conformers of each cluster.

Supplementary Table S2: Pendry reliability factors, R_P, comparison for the low and high wavenumber (low/high) IR signal of FPS-selected conformers of cluster **7** with IR signals of representative conformers of all other clusters. $\langle R_P \rangle$ values denote average values along with their standard deviation in the absence of the representative IR signal of cluster **7**. The "R_P to **7**" column denotes the R_P of the FPS-selected conformers to the representative IR signal of the cluster **7**. Note that smaller R_P values indicate a better match.

FPS conformer from **7**	$\langle R_P \rangle$ to all other representatives (low/high)	R_P to representative of **7** (low/high)
1	0.73 ± 0.02 / 0.92 ± 0.03	0.66 / 0.85
2	0.75 ± 0.01 / 0.94 ± 0.01	0.70 / 0.87
3	0.71 ± 0.03 / 0.95 ± 0.03	0.65 / 0.81
4	0.74 ± 0.05 / 0.94 ± 0.02	0.68 / 0.89
5	0.78 ± 0.03 / 0.96 ± 0.04	0.72 / 0.90
6	0.80 ± 0.04 / 0.98 ± 0.02	0.75 / 0.95
7	0.78 ± 0.04 / 0.97 ± 0.01	0.74 / 0.95
8	0.73 ± 0.03 / 0.95 ± 0.01	0.69 / 0.94
9	0.70 ± 0.05 / 0.91 ± 0.02	0.64 / 0.89
10	0.74 ± 0.03 / 0.93 ± 0.02	0.67 / 0.90

H-bonding and amide I IR peak shift

Supplementary Table S3: IR shift of amide I peak of PAMM representative conformations with respect to IRMPD experimental spectrum.

Cluster	w%	Amide I peak shift (cm^{-1})
**1**	13%	3.6
**2**	4.6%	10.1
**3**	9.8%	13.8
**4**	14.2%	10.2
**5**	20.1%	2.3
**6**	2.5%	23.2
**7**	29.3%	3.1
**8**	0.2%	18.2
**9**	6.3%	28.6

Supplementary references

(S1) Ghassabi Kondalaji, S.; Khakinejad, M.; Tafreshian, A.; J. Valentine, S. Comprehensive Peptide Ion Structure Studies Using Ion Mobility Techniques: Part 1. An

Advanced Protocol for Molecular Dynamics Simulations and Collision Cross-Section Calculation. *Journal of The American Society for Mass Spectrometry* **2017**, *28*, 947–959.

(S2) Re, S.; Watabe, S.; Nishima, W.; Muneyuki, E.; Yamaguchi, Y.; MacKerell, A. D.; Sugita, Y. Characterization of Conformational Ensembles of Protonated N-glycans in the Gas-Phase. *Scientific Reports* **2018**, *8*, 1644.

(S3) Kondalaji, S. G.; Khakinejad, M.; Valentine, S. J. Comprehensive Peptide Ion Structure Studies Using Ion Mobility Techniques: Part 3. Relating Solution-Phase to Gas-Phase Structures. *Journal of the American Society for Mass Spectrometry* **2018**, *29*, 1665–1677.

(S4) Hockney, R.; Goel, S.; Eastwood, J. Quiet high-resolution computer models of a plasma. *Journal of Computational Physics* **1974**, *14*, 148–158.

(S5) Berendsen, H.; van Gunsteren, W. H.J.C. Berendsen and W.F. van Gunsteren Practical Algorithms for Dynamic Simulation , in. *Molecular-Dynamics Simulation of Statistical Mechanical Systems* **1986**, 43–65.

(S6) Maier, J. A.; Martinez, C.; Kasavajhala, K.; Wickstrom, L.; Hauser, K. E.; Simmerling, C. ff14SB: Improving the Accuracy of Protein Side Chain and Backbone Parameters from ff99SB. *Journal of Chemical Theory and Computation* **2015**, *11*, 3696–3713.

(S7) Patriksson, A.; Van Der Spoel, D. A temperature predictor for parallel tempering simulations. *Physical Chemistry Chemical Physics* **2008**, *10*, 2073–2077.

(S8) Bussi, G.; Donadio, D.; Parrinello, M. Canonical sampling through velocity rescaling. *J. Chem. Phys* **2007**, *126*, 14101.

(S9) Neese, F. The ORCA program system. *WIREs Computational Molecular Science* **2012**, *2*, 73–78.

(S10) Becke, A. D. Density-functional exchange-energy approximation with correct asymptotic behavior. *Phys. Rev. A* **1988**, *38*, 3098–3100.

(S11) Perdew, J. P. Density-functional approximation for the correlation energy of the inhomogeneous electron gas. *Phys. Rev. B* **1986**, *33*, 8822–8824.

(S12) Weigend, F.; Ahlrichs, R. Balanced basis sets of split valence, triple zeta valence and quadruple zeta valence quality for H to Rn: Design and assessment of accuracy. *Physical Chemistry Chemical Physics* **2005**, *7*, 3297.

(S13) Grimme, S.; Antony, J.; Ehrlich, S.; Krieg, H. A consistent and accurate ab initio parametrization of density functional dispersion correction (DFT-D) for the 94 elements H-Pu. *The Journal of Chemical Physics* **2010**, *132*, 164117.

(S14) Laury, M. L.; Boesch, S. E.; Haken, I.; Sinha, P.; Wheeler, R. A.; Wilson, A. K. Harmonic vibrational frequencies: Scale factors for pure, hybrid, hybrid meta, and double-hybrid functionals in conjunction with correlation consistent basis sets. *Journal of Computational Chemistry* **2011**, *32*, 2339–2347.

(S15) Glielmo, A.; Husic, B. E.; Rodriguez, A.; Clementi, C.; Noé, F.; Laio, A. Unsupervised Learning Methods for Molecular Simulation Data. *Chemical Reviews* **2021**, *121*, 9722–9758.

(S16) Bolhuis, P. G.; Dellago, C. Trajectory-Based Rare Event Simulations. **2010**, 111–210.

(S17) Dellago, C.; Bolhuis, P. G.; Geissler, P. L. In *Lecture Notes in Physics*; Ferrario, M., Ciccotti, G., Binder, K., Eds.; Springer Berlin Heidelberg: Berlin, Heidelberg, 2006; Vol. 703; pp 349–391.

(S18) Rupp, M.; Tkatchenko, A.; Müller, K.-R.; von Lilienfeld, O. A. Fast and Accurate Modeling of Molecular Atomization Energies with Machine Learning. *Physical Review Letters* **2012**, *108*, 058301.

(S19) Sadeghi, A.; Ghasemi, S. A.; Schaefer, B.; Mohr, S.; Lill, M. A.; Goedecker, S. Metrics for measuring distances in configuration spaces. *The Journal of Chemical Physics* **2013**, *139*, 184118.

(S20) Ceriotti, M. Unsupervised machine learning in atomistic simulations, between predictions and understanding. *The Journal of Chemical Physics* **2019**, *150*, 150901.

(S21) Tribello, G. A.; Gasparotto, P. Using Dimensionality Reduction to Analyze Protein Trajectories. *Frontiers in Molecular Biosciences* **2019**, *6*, 46.

(S22) Helfrecht, B. A.; Gasparotto, P.; Giberti, F.; Ceriotti, M. Atomic motif recognition in (bio) polymers: Benchmarks from the protein data bank. *Frontiers in molecular biosciences* **2019**, *6*, 24.

(S23) Doerr, S.; Ariz-Extreme, I.; Harvey, M. J.; De Fabritiis, G. Dimensionality reduction methods for molecular simulations. **2017**,

(S24) Würger, T.; Mei, D.; Vaghefinazari, B.; Winkler, D. A.; Lamaka, S. V.; Zheludkevich, M. L.; Meißner, R. H.; Feiler, C. Exploring structure-property relationships in magnesium dissolution modulators. *npj Materials Degradation* **2021**, *5*.

(S25) Fiorin, G.; Klein, M. L.; Hénin, J. Using collective variables to drive molecular dynamics simulations. *Molecular Physics* **2013**, *111*, 3345–3362.

(S26) De, S.; Bartók, A. P.; Csányi, G.; Ceriotti, M. Comparing molecules and solids across structural and alchemical space. *Physical Chemistry Chemical Physics* **2016**, *18*, 13754–13769.

(S27) Bartók, A. P.; Kondor, R.; Csányi, G. On representing chemical environments. *Physical Review B* **2013**, *87*, 184115.

(S28) De, S.; Bartók, A. P.; Csányi, G.; Ceriotti, M. Comparing molecules and solids across structural and alchemical space. *Physical Chemistry Chemical Physics* **2016**, *18*, 13754–13769.

(S29) Musil, F.; Grisafi, A.; Bartók, A. P.; Ortner, C.; Csányi, G.; Ceriotti, M. Physics-inspired structural representations for molecules and materials. *Chemical Reviews* **2021**, *121*, 9759–9815.

(S30) Helfrecht, B. A.; Cersonsky, R. K.; Fraux, G.; Ceriotti, M. Structure-property maps with Kernel principal covariates regression. *Machine Learning: Science and Technology* **2020**, *1*, 045021.

(S31) Musil, F.; Grisafi, A.; Bartók, A. P.; Ortner, C.; Csányi, G.; Ceriotti, M. Physics-Inspired Structural Representations for Molecules and Materials. *Chemical Reviews* **2021**, *121*, 9759–9815.

(S32) Musil, F.; De, S.; Yang, J.; Campbell, J. E.; Day, G. M.; Ceriotti, M. Machine learning for the structure–energy–property landscapes of molecular crystals. *Chemical Science* **2018**, *9*, 1289–1300.

(S33) De, S.; Musil, F.; Ingram, T.; Baldauf, C.; Ceriotti, M. Mapping and classifying molecules from a high-throughput structural database. *Journal of Cheminformatics* **2017**, *9*, 6.

(S34) Ceriotti, M.; Tribello, G. A.; Parrinello, M. Simplifying the representation of complex free-energy landscapes using sketch-map. *Proceedings of the National Academy of Sciences* **2011**, *108*, 13023–13028.

(S35) Gasparotto, P.; Bochicchio, D.; Ceriotti, M.; Pavan, G. M. Identifying and tracking defects in dynamic supramolecular polymers. *The Journal of Physical Chemistry B* **2019**, *124*, 589–599.

(S36) Gasparotto, P.; Fischer, M.; Scopece, D.; Liedke, M. O.; Butterling, M.; Wagner, A.; Yildirim, O.; Trant, M.; Passerone, D.; Hug, H. J., et al. Mapping the Structure of Oxygen-Doped Wurtzite Aluminum Nitride Coatings from Ab Initio Random Structure Search and Experiments. *ACS Applied Materials & Interfaces* **2021**, *13*, 5762–5771.

(S37) de Marco, A. L.; Bochicchio, D.; Gardin, A.; Doni, G.; Pavan, G. M. Controlling exchange pathways in dynamic supramolecular polymers by controlling defects. *ACS nano* **2021**, *15*, 14229–14241.

(S38) Giberti, F.; Tribello, G. A.; Ceriotti, M. Global Free-Energy Landscapes as a Smoothly Joined Collection of Local Maps. *Journal of Chemical Theory and Computation* **2021**, *17*, 3292–3308.

(S39) Gasparotto, P.; Ceriotti, M. Recognizing molecular patterns by machine learning: An agnostic structural definition of the hydrogen bond. *The Journal of Chemical Physics* **2014**, *141*, 174110.

(S40) Gasparotto, P.; Meißner, R. H.; Ceriotti, M. Recognizing Local and Global Structural Motifs at the Atomic Scale. *Journal of Chemical Theory and Computation* **2018**, *14*, 486–498.

(S41) Ceriotti, M.; Tribello, G. A.; Parrinello, M. Demonstrating the Transferability and the Descriptive Power of Sketch-Map. *Journal of Chemical Theory and Computation* **2013**, *9*, 1521–1532.

(S42) Westerlund, A. M.; Delemotte, L. InfleCS: Clustering Free Energy Landscapes with

Gaussian Mixtures. *Journal of Chemical Theory and Computation* **2019**, *15*, 6752–6759.

(S43) Noé, F.; Clementi, C. Kinetic distance and kinetic maps from molecular dynamics simulation. *Journal of chemical theory and computation* **2015**, *11*, 5002–5011.

(S44) Noé, F.; De Fabritiis, G.; Clementi, C. Machine learning for protein folding and dynamics. *Current opinion in structural biology* **2020**, *60*, 77–84.

(S45) Martens, J.; Berden, G.; Gebhardt, C. R.; Oomens, J. Infrared ion spectroscopy in a modified quadrupole ion trap mass spectrometer at the FELIX free electron laser laboratory. *Review of Scientific Instruments* **2016**, *87*, 103108.

(S46) Bakker, J. M.; Besson, T.; Lemaire, J.; Scuderi, D.; Maître, P. Gas-Phase Structure of a π-Allyl-Palladium Complex: Efficient Infrared Spectroscopy in a 7 T Fourier Transform Mass Spectrometer. *The Journal of Physical Chemistry A* **2007**, *111*, 13415–13424.

(S47) Berden, G.; Derksen, M.; Houthuijs, K. J.; Martens, J.; Oomens, J. An automatic variable laser attenuator for IRMPD spectroscopy and analysis of power-dependence in fragmentation spectra. *International Journal of Mass Spectrometry* **2019**, *443*, 1–8.

(S48) Athokpam, B.; Ramesh, S. G.; McKenzie, R. H. Effect of hydrogen bonding on the infrared absorption intensity of OH stretch vibrations. *Chemical Physics* **2017**, *488-489*, 43–54.
