# Supplementary material for: Reconstructing the infrared spectrum of a peptide from representative conformers of the full canonical ensemble
Source: Commun Chem. 2023 Mar 3;6:46. doi: 10.1038/s42004-023-00835-3 (PMC9984374; doi:10.1038/s42004-023-00835-3)
Supplement: Supplementary file 2 — Description of Additional Supplementary Files [file 42004_2023_835_MOESM2_ESM.pdf]

Description of Additional Supplementary Files

File name: Supplementary Data

Description: Cartesian coordinates of DFT geometry optimized conformers of all clusters as well as the initial and final conformers of REMD simulation
